# Nomenclatural Remarks and Typifications in the Genus *Olea* L. (*Oleaceae*)

**DOI:** 10.3390/plants15020185

**Published:** 2026-01-07

**Authors:** P. Pablo Ferrer-Gallego, Jacek Wajer, Raúl Ferrer-Gallego

**Affiliations:** 1 Servicio de Vida Silvestre y Red Natura 2000, Centro para la Investigación y Experimentación Forestal (CIEF), Generalitat Valenciana, Avda. Comarques del País Valencià 114, 46930 Quart de Poblet, Valencia, Spain; 2 Bodega Ferrer-Gallego, 46311 Jaraguas, Valencia, Spain; 3 Natural History Museum, Cromwell Road, London SW7 5BD, UK; j.wajer@nhm.ac.uk; 4 Department of Ecology, Desertification Research Centre (Spanish National Research Council-University of Valencia and Valencian Government), 46113 Moncada, Valencia, Spain

**Keywords:** agriculture, commercial crop, economic botany, lectotype, Mediterranean Basin, oleaster, acebuche, Philip Miller, *Olea europaea*

## Abstract

The typification of five names in the genus *Olea* (Oleaceae), viz. *O. europaea* subsp. *cerasiformis*, *O. europaea* var. *maderensis*, *O. cuspidata*, *O. laperrinei*, and *O. sylvestris* is discussed. These taxa are currently classified within *O. europaea* at the infraspecific rank. The designation of the types is based on the consultation of original material conserved in several herbaria and the literature cited in the respective protologues. The name *O. europaea* subsp. *cerasiformis* is lectotypified from a specimen preserved at FI. The name *O. europaea* var. *maderensis* (also named *O. europaea* subsp. *maderensis* or *O. maderensis*, and currently treated as a synonym of *O. europaea* subsp. *cerasiformis*) is lectotypified from a specimen collected by Lowe in Madeira and preserved at BM. The name *O. cuspidata* (currently *O. europaea* subsp. *cuspidata*) is lectotypifed from a specimen collected in India and preserved at K. The name *O. laperrinei* (currently *O. europaea* subsp. *laperrinei*) is lectotypifed from a specimen preserved at MPU and collected in the Sahara Desert (Ahaggar Mountains, Algeria). Finally, the name *O. sylvestris* (currently *O. europaea* var. *sylvestris*), wild olive, also named oleaster or acebuche (Spanish language), a wild relative of the olive tree, is lectotypified on a Miller specimen preserved at BM.

## 1. Introduction

The genus *Olea* L. (*Oleaceae*) contains 33 species and 9 subspecies of evergreen trees and shrubs, extant throughout Africa, Europe, Asia and Oceania [1,2,3,4]. Systematic studies have recognized up to 3 subgenera within *Olea* (subg. *Olea*, *Paniculatae* (Knobl.) P.S. Green, and *Tetrapilus* (Pers.) P.S. Green). Additionally, within *O.* subg. *Olea*, two sections are recognized (sect. *Olea* and sect. *Ligustroides* (Lour.) P.S. Green), with the former being exclusively reserved for the taxonomic complex of *O. europaea* L., which includes up to 6 subspecies (subsp. *europaea*, subsp. *cuspidata* (Wall ex G. Don) Cif., subsp. *laperrinei* (Batt. & Trab.) Cif., subsp. *maroccana* (Greuter & Burdet), subsp. *cerasiformis* G. Kunkel & Sunding, and subsp. *guanchica* [1,4,5,6,7,8,9,10,11,12,13,14,15,16,17]. The subsp. *europaea* is further subdivided into two taxonomic varieties: var. *sylvestris* (Mill.) Lehr, also named oleaster or wild olive, which comprises the wild forms of the olive tree [18,19,20,21], and var. *europaea*, which encompasses around 1000 cultivated forms [4,12,18,22,23,24]. The olive tree is part of a broad taxonomic complex that extends from Africa to Asia [2,3,25,26].

The olive tree is probably the most symbolically rich tree in the Mediterranean basin [27]. In the past (and in the present), it has been a symbol of friendship and peace among nations. As early as the 7th century BC, winners of the Olympic Games were awarded a wreath of olive branches [28]. Olives are nowadays considered a condiment or an appetizer product, but they were a true food source in antiquity. The fruits of certain cultivars can be eaten as they are when ripe, but most require debittering. This process serves to remove a bitter polyphenol, oleuropein [27,29,30,31]. Moreover, the olive tree gained enormous economic and cultural significance as a source of oil, since olive oil was not only an essential food, but also a key resource for lighting, medicine, religious rituals, and trade throughout the Mediterranean world [32,33]. Today, the olive remains highly important [34,35], as it is the most economically significant oil-producing tree crop in temperate regions worldwide, with approximately 11.5 million hectares currently under cultivation [36].

From the perspective of the nomenclature of this relevant group of plants, effective typifications exist for three names:

(1) The lectotype of the Linnaean name *O. europaea* is the specimen preserved at Herb. Clifford: 4, *Olea* 1α BM (barcode BM000557513), designated by Green & Wickens (1989: 294) [1].
(2) The holotype of *O. europaea* subsp. *guanchica* is the specimen at MA with barcode MA643248 (see below), with an isotype at MJG. This material was collected in La Gomera, Canary Islands [37,38,39] sub *O. cerasiformis* Rivas Mart. & del Arco, as a replacement name of *O. europaea* subsp. *guanchica* P. Vargas et al. [6].
(3) The holotype of *O. maroccana* Greuter & Burdet is somewhat cryptic. *Olea maroccana* is a replacement name of *O. salicifolia* Barbéro, Benabid, Quézel, Rivas Mart. & A. Santos in Doc. Phytosoc., ser. 2, 6: 319 (1982), nom. illeg.; an illegitimate later homonym (see *ICN* Art. 53.1) of *O. salicifolia* Wall. ex G. Don fil. Gen. Hist. 4: 48. 1837. The name is currently accepted as *O. europaea* subsp. *maroccana* (Greuter & Burdet) P. Vargas et al. [6,7,12]. The holotype was indicated in the protologue as “*in ditione Ida ou Tanane*, *regno maroccano*, *prope Tamrhaght 250 m. In herbario facultatis-Saint-Jérôme*, *Marseille*” [In the region of Ida or Tanane, Moroccan kingdom, near Tamrhaght, 250 m. In the herbarium of the Faculty of Saint-Jérôme, Marseille] [40]. Later, Médail and co-workers [7] mentioned “S.W. Morocco, Ida-ou-Tanane, near Tamrhakht, P. Quézel, iv.1982 (MARSSJ TYPUS) (inflorescence)”. MARSSJ is the herbarium code of the herbarium at Université Paul Cézanne, in Mirabeau, France.

However, five names, *O. europaea* subsp. *cerasiformis*, *O. europaea* var. *maderensis*, *O. cuspidata* (currently *O. europaea* subsp. *cuspidata*, *O. laperrinei* (currently *O. europaea* subsp. *laperrinei*), and *O. sylvestris* Mill. (currently *O. europaea* var. *sylvestris*), are untypified and are examined here. The types of these names are discussed in this work as a foundation for the taxonomic study, providing clarity and consistency in olive taxonomy and enabling more accurate identification and comparison of specimens across regions and collections.

## 2. Results and Discussion

### 2.1. Biological and Nomenclatural Background

The molecular finding has supported the *O. europaea* complex as a characteristic example of a tropical African lineage, of which three different evolutionary units have later colonized several distinct areas, notably North Africa and the Mediterranean Basin [2,3,7,9,10,12]. The migration of the ancestral *Olea* probably occurred prior to the desertification of the Sahara and dates at least from the Pliocene [41,42,43].

The olive tree (*O. europaea* subsp. *europaea*) is the most iconic tree of the Mediterranean basin, with origins linked to the rise in some of the world’s oldest civilizations, dating back at least six thousand years [27,32,33,44,45,46,47]. The olive, probably the first domesticated fruit tree, is one of the most important classical Mediterranean crops both economically and culturally. Archaeobotanical findings of olive stones in human habitats date back to ca. 780,000 years BP, Acheulian Gesher Benot Ya’akov, Hula alley [48], and ca. 23,000 BP Epipalaeolithic Ohalo II, Sea of Galilee, Israel [49,50]. Later Neolithic and Chalcolithic sites throughout the Mediterranean Basin, including the Levant, also contain olive remains in quantities that indicate collection from wild trees inhabiting the area [47].

The origin and current selection of the cultivated olive tree by humans is not fully understood, but some studies suggest it may have originated in the eastern Mediterranean region [51,52] or in the region adjacent to the western Nile Delta [53]. The cultivated plant, belonging to the type subspecies, is very sparsely represented in natural wild populations and should be regarded as an ancient cultivated form. Genetic and phenotypic evidence suggest the existence of indigenous populations of wild olive, var. *sylvestris*, along the Carmel coast in southern Levant [54]. In addition, other populations have been documented throughout the Mediterranean Basin [17,55,56]. On the other hand, wild forms of this species, known as oleaster or acebuche (Spanish language), and also named as *O. europaea* var. *sylvestris* (Mill.) Lehr, as well as feral forms of cultivated varieties, are known [4,18,20,55,57,58,59].

The wild olive is a woody plant that characterizes the Mediterranean vegetation landscape, where it is an emblematic component of the maquis vegetation. It colonizes coastal areas and even hill stations, where it prefers sites with a southern exposure, including those that are dry and xeric. Regarding the phytosociological interpretation of mature forest expressions of oleaster distributed throughout the Mediterranean basin, many associations have already been described; in particular, they regard North Africa [20,39,60,61].

Regarding the origin of currently cultivated varieties, it has traditionally been thought that the selection and vegetative propagation of the most suitable spontaneous forms (var. *sylvestris*) led to the creation of the first cultivated varieties [54,56,62,63,64]. However, it is not entirely clear that all known cultivars originated this way, as they come both from wild olives and from crosses between these and other cultivars [19,57,65]. The relationships between cultivated olive (var. *europaea*) and wild olive (var. *sylvestris*) are not clear, making it difficult to distinguish between plants that have become feral from cultivated olive trees and truly wild ones, even with the use of molecular and phenotypic markers that assist in the identification and more precise diagnosis of the taxa [55,66]. However, some genetic studies have revealed the origin of certain populations, documenting the native range of populations growing outside the Mediterranean region, such as in northern Spain [38,67].

The oleaster has a circum-Mediterranean distribution [17,20,38,61]. Generally, acebuche (Spanish language) can be differentiated from cultivated forms by its smaller fruits and leaves, sometimes nearly round, and its lower branches being spiny, compared to the generally larger fruits and leaves of var. *europaea*. However, because stone morphology and the size of wild and domestic olives overlap considerably, current research on pit morphology alone can not be used to identify domesticated olives [47]. Recently, the first step has been made towards distinguishing between spontaneous (uncultivated) and cultivated varieties based on seed morphotypes [58,59]. According to Bolòs & Vigo [68], some fruit and leaf measurements can be useful for field identification. For example, var. *sylvestris* typically has fruits no larger than 1–2 cm and leaves 1–4(7) × 0.4–2.2 cm, compared to the average measurements for var. *europaea*, with fruits 2–3.5 cm and leaves 4–8 × 0.6–2 cm [4,55,69,70].

The olive tree is one of the most complex cases in the study of fruit tree domestication [32,33,71]. Undoubtedly, the wild olive tree has played a very important role in the expansion of this crop and in the improvement and diversity of the crop. The numerous uses of both cultivated and wild olives as sources of food, wood, forage, and more explain the widespread expansion of olive groves. According to some authors, olive cultivation has led to the creation of nearly 2000 cultivars in the Mediterranean region [72].

Olive domestication is characterized by the vegetative propagation of the most valuable genotypes [27], selected for their agronomic value, primarily based on better fruit set, larger olives, and higher oil content, as well as their ability to thrive in anthropogenic environments. The ease with which these genotypes can be propagated vegetatively through cuttings or grafting has also influenced their selection. Grafting cultivated olive varieties onto local oleaster genotypes has been a widely practiced technique, used since ancient Greece and Rome [73,74,75]. This type of grafting aims to produce productive varieties with more resistant rootstocks [76].

Unlike other fruit crops, such as grapevines or apples [77,78,79], the production of rootstocks is poorly documented in olives [80,81]. However, Barazani and co-authors [74] demonstrated that certain genotype combinations between scions and rootstocks do not follow a random geographical distribution. This suggests that producers may have selected specific combinations in certain areas of the Mediterranean, possibly to improve characteristics such as oil quality or drought tolerance [66].

The typification of the name *Olea sylvestris* is a topic of debate in this paper. The absence of a formal typification for such a widespread species is problematic, as it leads to instability, particularly given the ongoing uncertainties surrounding its taxonomy. This article aims to address and resolve this issue.

On the other hand, regarding the recognized taxa with subspecies rank, *O. europaea* subsp. *maroccana* is distributed in the Agadir Mountains (western Sahara region). According to molecular analysis [4,5,8], the populations of *O. europaea* from Agadir are more closely related to populations from the Canary Islands (subsp. *cerasiformis*) than to those from the Hoggar mountains (subsp. *maroccana*). Considering these results, the populations of *O. europaea* from the Agadir mountains have been considered at the subspecific rank. *Olea maroccana* Greuter & Burdet was published as a replacement name of *O. salicifolia* Barbéro, Benabid, Quézel, Rivas Mart. & A. Santos [40], an illegitimate later homonym (see *ICN* Art. 53.1) of *O. salicifolia* Wall. ex G. Don fil. Gen. Hist. 4: 48. 1837. The epithet “*maroccana*” is currently used at the subspecific rank, *O. europaea* subsp. *maroccana* (Greuter & Burdet) [6].

Finally, *O. europaea* subsp. *laperrinei* is distributed in the Saharan massifs, and *O. europaea* subsp. *cuspidata* has a broad distribution ranging from South Africa to southern Egypt and from Arabia to northern India and southwestern China [4,9,12,20]. *Olea europaea* subsp. *guanchica* is endemic to the Canary Islands [4,7,13,15,82,83]. This taxon was also proposed at a specific rank as *Olea cerasiformis* Rivas Mart. & del Arco [39]. In contrast, the name *O. europaea* subsp. *cerasiformis* refers to the Madeiran endemic taxon [4,13]. It has occasionally been referred to as *O. europaea* subsp. *maderensis* (Lowe) O.E. Erikss., A. Hansen & Sunding, and has also been treated at a specific rank as *O. maderensis* (Lowe) Rivas Mart. & del Arco [6,7,12,39,84,85,86,87].

### 2.2. Typification of the Names

#### 2.2.1. *Olea europaea* subsp. *cerasiformis*, *O. europaea* var. *maderensis*, and *O. europaea* subsp. *guanchica*

##### *Olea europaea* subsp. *cerasiformis*

The nomenclature of the two Macaronesian endemic taxa is complex. The Canarian endemic plant has been considered either a species (i.e., *O. cerasiformis*) [39] or a subspecies (i.e., *O. europaea* subsp. *guanchica*) [4,6,15]. The Madeiran endemic taxon, on the other hand, has been generally treated at infraspecific ranks (*O. europaea* var. *maderensis* and *O. europaea* subsp. *maderensis*) [85] or, more recently, as a species (*O. maderensis*) [39]. This taxon is sometimes referred to as *O. europaea* subsp. *cerasiformis* Webb & Berth. ex Kunkel & Sunding) [6,88]. At a specific rank, the epithet “*cerasiformis*”, proposed by Webb & Berthelot [89] for an illegitimate name at varietal rank (*O. europaea* β [var.] *cerasiformis*), as noted above, has also been used [39].

*Olea europaea* β [var.] *cerasiformis* Webb & Berth. (in Hist. Nat. Îles Canaires 3(2,3): 162. 1845) [89] is an illegitimate and superfluous name because its protologue includes the name *O. europaea* var. *maderensis* Lowe [90], which should have been adopted, or whose epithet should have been adopted (see *ICN* Art. 52.1 and 52.2). Webb & Berthelot mentioned [89]: “*Olea europæa/β cerasiformis. Frutex*, *ramis debilibus foliis elongato-linearibus*, *mucronulatis*, *subtùs subglabris*, *serè concoloribus*, *racemis compositis divaricatis*, *drupis parvis subrotundis./Oleæ europææ sp. valdè simile Oleæ glabellae. R. Br. Verzeichn. d. auf. Mad. wildwachs. Pfl. in Buch*, *Can.*, *pag. 192./Olea europæa var. maderensis. Lowe*, *Nov. fl. mad.*, *pag. 15 […]/Obs. In foliis var. β Æcidium aut ejectatio quædm oritu alba nitens margaritacea. -Hanc in foliis nullius Oleastri Europæi animadvertit doctissimus atque harum expertissimus rerum Montagneus*, *unde forma forsam nova. Hæc quoque manna forsitan de quâ l. c. apud cl. Loweum agitur.*” [*Olea europaea*, β *cerasiformis*. A shrub, with slender branches and elongated, linear leaves, mucronulate, somewhat glabrous beneath, becoming nearly concolorous when mature; inflorescences in compound, spreading racemes; drupes small and somewhat rounded. *Olea europaea* sp. very similar to *Olea glabella* R. Br. (Verzeichn. d. auf Mad. wildwachs. Pfl. in Buch, Can., p. 192.) *Olea europaea* var. *maderensis*. Lowe, Nov. fl. mad., p. 15 […] Observation. On the leaves of variety β, an *Æcidium* or a kind of excrescence arises as white, shining, pearly spots. This was observed on the leaves of no European oleaster by the most learned and experienced authority in these matters, Montagneus, from which perhaps a new form may originate. This may also be the “manna” mentioned in the same place in the work of the distinguished Lowe].

However, Webb & Berthelot’s [89] illegitimate name “*O. europaea* var. *cerasiformis*” provides the final epithet for names of a taxon published at other ranks (i.e., subspecific and specific) [4,6,39] that are in continuous use for a Madeiran wild olive. In this sense, the epithet “*cerasiformis*” was later used by Kunkel & Sunding (in Kunkel [88]) for a taxon at the subspecific rank.

Kunkel & Sunding (in Kunkel [88]) mentioned “*Olea europaea* subsp. *cerasiformis* (Webb & Berth.) Kunkel & Sunding, comb. nov./*O. europaea* L. var. *cerasiformis* Webb & Berth., Phytogr. Canar. 3: 162 (1845). ?end. canar.”. However, it cannot be treated as a combination since Webb and Berthelot’s name is illegitimate, but it can be treated as a new name based on their publication, in which a description of the plant appears along with a reference to a specific gathering. Therefore, the original material for this name (*Olea europaea* subsp. *cerasiformis* G. Kunkel & Sunding) should be sought within the context of Webb & Berthelot’s publication [89], cited by Kunkel & Sunding (in Kunkel [88]). These authors (Webb & Berthelot [89]) mentioned: “*Olea europaea/β cerasiformis. Frutex*, *ramis debilibus foliis elongato-linearibus*, *mucronulatis*, *subtùs subglabris*, *serè concoloribus*, *racemis compositis divaricatis*, *drupis parvis subrotundis.* […] */Hab. […] Var. β in altis montibus Canariae invenit Despréaux.*” [*Olea europaea* β *cerasiformis*. A shrub, with slender branches and elongated, linear, mucronulate leaves, somewhat glabrous beneath, becoming nearly concolorous when mature; inflorescences in compound, spreading racemes; drupes small and somewhat rounded. Habitat. […] Variety β was found in the high mountains of the Canary Islands by Despréaux].

We have located only one specimen that directly corresponds to the gathering cited by Jean-Marie Despréaux in the Philip Barker Webb herbarium at FI. The sheet, with barcode FI000164, bears a stem with leaves and flowers, and a handwritten label, annotated as: “N° 410./Varieta a feuilles congestes/[illegible]/La cumbre de Canarias/au milieu des rochers” (see below). Jean-Marie or Louis Despréaux Saint-Sauveur (1794–1843) was a French botanist who explored the Canary Islands. However, there is no evidence that he specifically traveled to Madeira in the available sources on his life and expeditions. His documented fieldwork is mainly concentrated in the Canary Islands after his participation in the scientific expedition to Morea (Greece).

We did not locate any additional original material for this name. The specimen FI000164 is part of the gathering cited by Webb & Berthelot in 1845 [89] and therefore represents the basis of the name *O. europaea* subsp. *cerasiformis*. As such, it appears to be the obligatory lectotype for the name. However, this material from the Canary Islands (most likely from Gran Canaria) may not correspond to the current concept of the Madeiran endemic taxon. Nevertheless, the names *O. europaea* subsp. *cerasiformis* and *O. europaea* subsp. *guanchica* could potentially be conspecific (their corresponding types originate from the Canary Islands), which would entail a proposal for conservation with a conserved type, a matter that is currently under detailed investigation. In this context, the name proposed by Rivas Martínez & del Arco [39] as *O. cerasiformis* for the Canary Islands plants, based on *O. europaea* subsp. *guanchica*, blocks the specific-rank recombination of Kunkel & Sunding’sname (see below). The study of the type material of *O. europaea* subsp. *cerasiformis* is essential to establish a clear unambiguous nomenclature in the application of the name of a taxon at the subspecies rank. This matter is currently under consideration.

##### *Olea europaea* var. *maderensis*

At present, the olive tree of the Madeira archipelago is known as *O. europaea* subsp. *cerasiformis* [4,6,15]. This taxon has been given other names throughout its history, which we examine below.

One of these names is *Olea europaea* var. *maderensis*. Green (2002: 98) [4] mentioned a specimen at “BM” as the “holotype” of the name: “*O. europaea* var. *maderensis* Lowe, Trans. Cambridge Philos. Soc. 4 (3): 537 (1838) [90]; Nov. Fl. Mader.: 15 (1838) & Man. Fl. Madeira 2: 21 (1872) [91]. Type: Madeira, *Masson* (holotype, BM)”. Although Lowe certainly mentioned in the protologue a specimen collected by Masson (see below), he did not indicate that it constituted the type material. Moreover, there are other specimens that may be considered as original material used by Lowe for the description of his species, so the Masson specimen at BM is not the only material available for the typification of this name. In addition, although the selection of the specimen at BM as lectotype would be appropriate, Green’s use of “holotype” cannot be corrected to a lectotypification because the phrase “here designated” (“*hic designatus*”) or an equivalent expression was not used, according to the International Code of Nomenclature (*ICN*) (Art. 7.11) [92].

The protologue of this name (Lowe, 1838) [90] includes a description in Latin “*foliis lineari-oblongis*, *angustis*, *mucronatis*, *integerrimis*, *utrique subconcoloribus s. inferne nudiusculis: drupis subglobosis*, *purpurascentibus*, *demum nigris*” [Linear-oblong leaves, narrow, pointed, entire, uniformly colored on both sides or slightly more bare on the underside; subglobose fruits, purplish in color, eventually black], followed by the provenance “*Hab. in rupibus apricis Maderæ*, *præsertim maritimis*” [It is found on sun-exposed rocks in Madeira, especially in the coastal areas], and the relevant comment “*Specimen in Herbario Banksiano*, *a cl. Masson olim lectum*, *sub nomine O. europææ a cl. R. Brown in “Von Buch’s Catalogue”*, *O. glabellæ Herb. Banks. (O. exasperatæ Jacq. Hort. Schoenbr. III. t. 1.) “valde simile” dicitur. Panicula vero terminali*, *ramisque tuberculatis hæc satis differe videtur*” [A specimen in the Banksian Herbarium, collected by Mr. Masson [in as undisclosed locality] and listed by Mr. R. Brown in “Von Buch’s Catalogue” under the name *O. europææ*, is very similar to *O. glabella* in Herb. Banks. (or *O. exasperata* Jacq. Hort. Schoenbr. III. t. 1.). However, the panicle is terminal, and the branches are tuberculate, which seems to differ quite a lot from this], see Lowe (1853) [93].

Robert Brown (1773–1858), Banks’s librarian and later the first keeper of the Banksian (later Botanical) Department of the British Museum, based his first comprehensive list of the Madeiran flora (now kept in the Special Collections library at the Natural History Museum in London) mostly on Masson’s collections housed at the BM but the collections made by Banks and Solander were also one of the obvious sources for his study [94]. The actual date when Brown prepared this list is unknown, but this floristic account remained unpublished until the works of Buch in 1825 [95] and Britten in 1904 [96]. It is noteworthy that Brown himself visited Madeira in 1801 en route to Australia on board HMS *Investigator* [97].

In Joseph Banks’s unpublished manuscripts relating to the Flora of Madeira, specifically in a small booklet written by Jonas Dryander (one of the curators of Banks’s collections) which is a handwritten list of Masson’s specimens from Madeira called “*Massonii Flora Maderensis*” we have found a clear reference to *O. europaea* L., which means that there used to be a specimen of this species collected by Masson in Madeira in the Banksian Herbarium. This also means that the specimen cited by Lowe in the protologue of *O. europaea* var. *maderensis* did exist.

**Figure 1:**
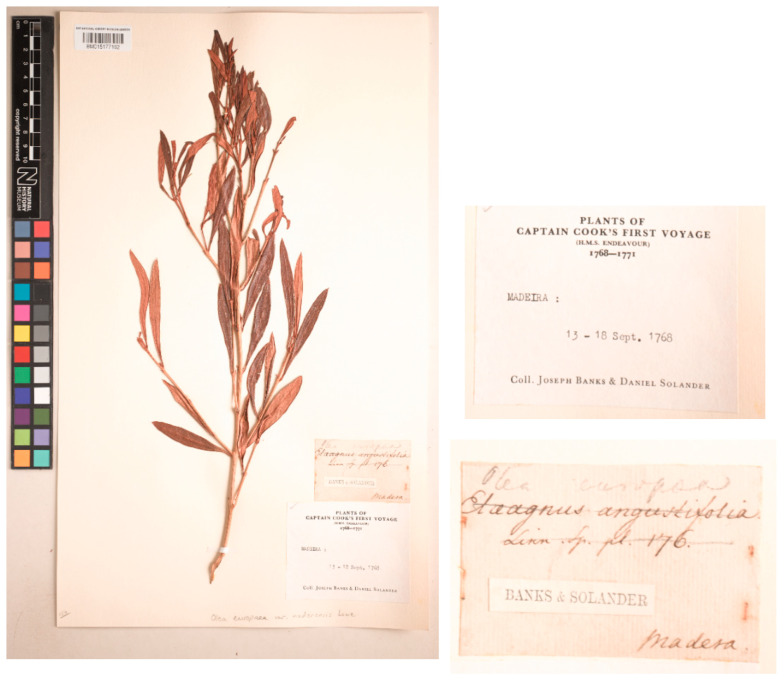
Specimen of *Olea europaea* var. *maderensis* Lowe collected in Madeira by Banks and Solander and preserved at BM, barcode BM015177192. Image courtesy of the herbarium BM, reproduced with permission.

A specimen in the General Herbarium at BM collected by Banks & Solander would have been available to Lowe, as we have no idea if he has examined it. The sheet barcoded BM015177192 contains a branch with leaves but no fruits, and a handwritten label: “*Olea europaea*/*Elaeagnus angustifolia*/Linn. Sp. pl. 176./Madeira” as well as a printed slip with the names “Banks & Solander”. This material comes from Captain Cook’s expedition, as indicated on a printed label also attached to the sheet: “Plants of/Captain Cook’s First Voyage/(H.M.S. Endeavour)/1768–1771/Madeira:/13–18 Sept. 1768/Coll. Joseph Banks & Daniel Solander.”. On the lower part of the sheet, the name is written in pencil directly on the sheet “*Olea europaea* var. *maderensis* Lowe” (see Figure 1). However, as noted by Santos-Guerra et al. [94], this specimen was not examined by Lowe for the *Flora of Madeira*.

**Figure 2:**
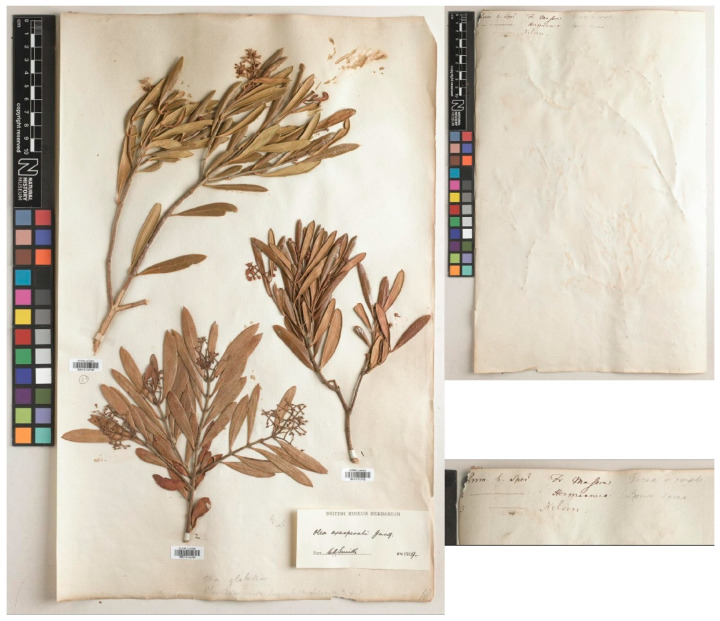
Sheet preserved in the Herbarium Banks at BM with three specimens of *O. exasperata* collected in the Cape of Good Hope. Masson’s gathering barcoded BM010763788; Hermann’s gathering barcoded BM010763789, and Nelson’s gathering barcoded BM010763790. Image courtesy of the herbarium BM, reproduced with permission.

On the other hand, Lowe (1838) [90] clearly indicated that a specimen in the Herb. Banks is very similar to *O. glabella* Banks ex Lowe (noted above) or *O. exasperata* Jacq., and this is relevant because there is a sheet of *O. exasperata* (which is a strictly South African taxon) from Herb. Banks on which Masson’s gathering from the Cape of Good Hope (from Madeira) (BM010763788) is mixed with two other collections from the Cape region by Hermann (BM010763789) and Nelson (BM010763790) (see Figure 2). However, this material belongs to a different species and therefore cannot be considered original for the name *Olea europaea* var. *maderensis*.

Von Buch’s publication includes a chapter on the plants of Madeira, which he himself attributes to R. Brown (Buch, 1825) [95]. Brown’s undated, handwritten list of the plants of Madeira, arranged by Linnaeus’s Sexual System, is still preserved at the BM Library (Banks MSS Ref. No. B. 14), and von Buch makes direct use of this manuscript, in many places copying it almost verbatim (see Britten, 1904) [96]. In von Buch (1825: 192) [95], the relevant text is as follows: “*OLEA europaea. Sp. valde simile Olea glabellae. Fructus parvi subrotund*, *stylo persistenti coronali*” [*Olea europaea*. A species very similar to *Olea glabella*. The fruits are small, somewhat rounded, crowned by a persistent style]. Thus, Lowe’s protologue is, in part, a direct transcription of Brown’s wording, although Lowe adds certain details—notably that there was a specimen in the Banks Herbarium included in Brown’s list as *O. europaea* (which is consistent, since Brown compiled his Madeira list by examining Banks’s specimens). This indicates that the phrase “*valde simile dicitur*” is simply R. Brown’s observation as transmitted through von Buch, expressing Brown’s view that the plant resembled *O. glabella*. It is therefore not an opinion or taxonomic judgment introduced by Lowe, but merely text copied from Von Buch’s publication. Consequently, the name *O. glabella* appears to have been first validated by Von Buch, not by Lowe, contrary to what is incorrectly implied in IPNI (see https://www.ipni.org/search?q=Olea%20glabella) (accessed on 15 January 2025).

**Figure 3:**
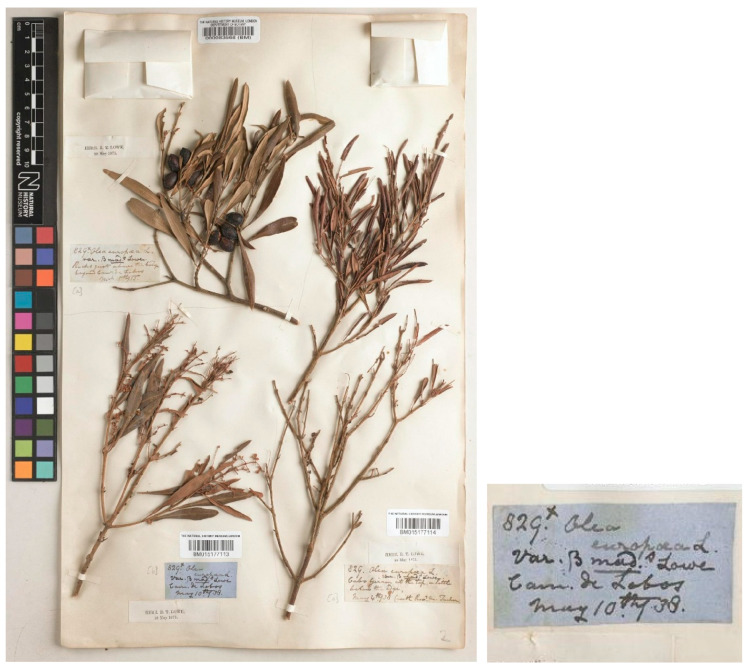
Specimens of *Olea europaea* var. *maderensis* Lowe collected in Madeira by Lowe and preserved at BM, barcodes BM000083568, BM015177113, collected near Câmara de Lobos in 1855 and 1838, respectively; and BM015177114 collected near Cabo Girão in 1838. The specimen BM015177113 is selected as the lectotype of the name *Olea europaea* var. *maderensis*. Image courtesy of the herbarium BM, reproduced with permission.

All these three gatherings from South Africa (i.e., BM010763788, BM010763789, and BM010763790), including the one by Masson, look remarkably similar to three gatherings of olives collected in Madeira by Lowe himself, which are filed at BM as *Olea europaea* (BM000083568, BM015177113, BM015177114) (Figure 3). Two of these specimens were collected by Lowe before he had read his paper at the Cambridge Philosophical Society on 28 May 1838 (mentioned at the beginning his paper, see Lowe (1838: 523 [“Read *May* 28, 1838”])), i.e., BM015177113 (collected near Câmara de Lobos (Madeira) on May 10, 1838) and BM015177114 (collected near Cabo Girão (Madeira) on May 4, 1838) (Figure 3). Both of these gatherings are also cited by Lowe in his much later publication, i.e., *A Manual Flora of Madeira* (Lowe, 1872) [91], as “*Olea europæa* L. […] *β. maderensis* Lowe […] Lowe Novit. (1838) p. 15 or 537 [90]. *O. europæa β. cerasiformis* WB. (1840) iii. 162. *O. europæa*, “Sp. valde simile *Oleæ glabellæ*” (scil. Herb. Banks. = *O. exasperata* Jacq.), “*Fructus parvi subrotundi stylo persistente coronati” Buch! Mad. List 192. no. 168*.” (see also Buch, 1825: 192) [95]. The specimen barcoded BM000083568, with leaves and fruits, was also collected by Lowe in Câmara de Lobos (Madeira), but with a date (March 15, 1855) much later than the publication of the protologue, so it cannot be considered as original material.

There is another sheet at BM belonging to a different Lowe collection. The sheet BM000083570 bears a branch with leaves but no fruits, and an original handwritten label by Lowe, which reads as follows: “225, f./(copy)/Olea europaea, L./Hermigua, Apr. 20./61. [i.e., collected on 20 April 1861]”. This sheet, with a specimen collected on the island of La Gomera, contains material belonging to this species, but it cannot be considered original material, as it was collected long after the publication of the protologue.

However, the two specimens collected by Lowe at the beginning of May 1838 (i.e., BM015177113 and BM015177114) could be treated as uncited elements of original material, and one of them should be chosen as a lectotype. We have not located any other material collected by Lowe in Madeira that could be considered as probable original material for the name *O. europaea* var. *maderensis*.

In conclusion, among the specimens analyzed above, barcoded BM015177113, BM015177114, BM000083570, and BM015177192, since it is not possible to demonstrate that the specimen BM015177192 was studied or cited by Lowe in his protologue, it does not have priority for the selection of the type. Lowe cites material collected by Masson and preserved in the Banks Herbarium, but unfortunately, we have not been able to locate it; such material would undoubtedly have been preferable for selection as the lectotype. On the other hand, the two specimens collected in May 1838, which may represent original material of Lowe, are very close in date to the publication of the protologue (28 May 1838) and were undoubtedly available to the author before its publication. Between these two specimens, we select the specimen BM015177113 as the lectotype of the name because it is the most complete and informative.

**Figure 4:**
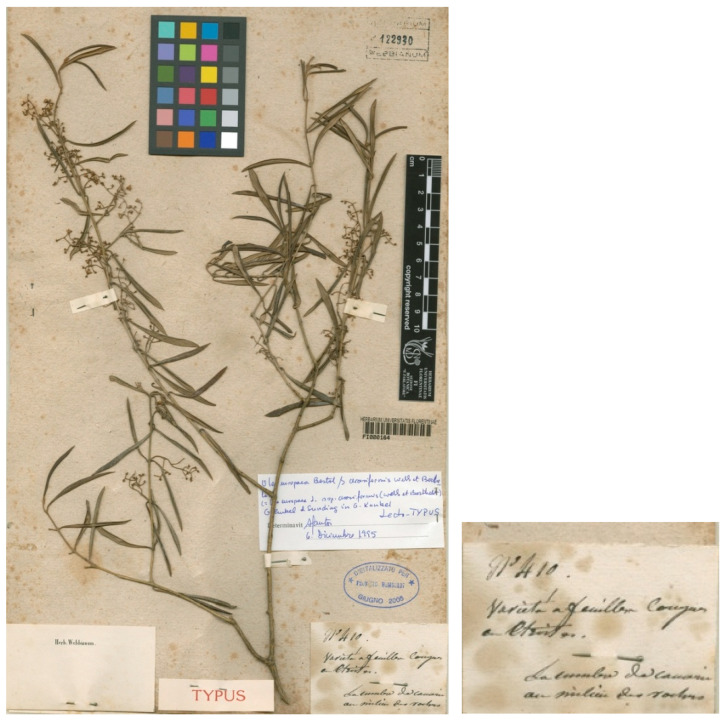
Lectotype of *Olea europaea* subsp. *cerasiformis* Webb & Berth. ex G. Kunkel & Sunding, FI barcode FI000164. Image courtesy of the herbarium FI, reproduced with permission.

***Olea europaea*** subsp. ***cerasiformis*** Webb & Berth. ex G. Kunkel & Sunding, Mongr. Biol. Canar. 3: 58. 1972–**Lectotype (designated here):** Spain, Gran Canaria, s.d., *J.-M. Despréaux 410*, FI (barcode FI000164). For an image of the original material, see Figure 4.

= *Olea europaea* var. *maderensis* Lowe in Trans. Cambridge Philos. Soc. 6: 537. 1838 ≡ *O. chrysophylla* var. *maderensis* (Lowe) A. Chev. in Rev. Bot. Appl. Agric. Trop. 28: 20. 1948 ≡ *O. europaea* subsp. *maderensis* (Lowe) O.E. Erikss., A. Hansen & Sunding in Fl. Macaronesia, ed. 2: 60. 1979 ≡ *O. maderensis* (Lowe) Rivas Mart. & del Arco in Itin. Geobot. 15: 705. 2002–**Lectotype (designated here):** Portugal, collected near Câmara de Lobos (Madeira) on May 10, 1838, *Lowe 829*, BM barcode BM015177113. For an image of the lectotype, see Figure 3.

##### *Olea europaea* subsp. *guanchica*

*Olea europaea* subsp. *guanchica* is a wild olive taxon native to the Canary Islands. It is considered endemic to the archipelago and represents the natural, non-cultivated olive lineage found especially in Tenerife, La Palma, El Hierro, and La Gomera [4,6]. The subspecies is morphologically and genetically distinct from both the widespread Mediterranean wild olive (*O. europaea* var. *sylvestris*) and the Madeiran wild olive (*O. europaea* subsp. *cerasiformis* sensu stricto). It typically grows as a shrub or small tree in dry scrublands and rocky slopes, forming part of thermophilous woodland remnants. Leaves tend to be smaller and narrower than those of cultivated olives, and the fruits are usually small, dark, and less fleshy [39,82,83].

Rivas-Martínez et al. [39] proposed the name *O. cerasiformis* as a replacement name (with the phrase “sp. nova”) based on the basionym and description of *O. europaea* subsp. *guanchica* [6] (that is the replaced synonym), and not on *Olea europaea* var. *cerasiformis* Webb & Berthel., legitimized by Kunkel & Sunding (in Kunkel [88]). Curently, the subspecies rank is accepted to account for the full generic and morphological diversity of *O. europaea*; however, some authors advocate for the species rank [40]. In this context, the most appropriate approach is to recognize the Canary Islands plant under the name *O. guanchica* (P. Vargas, J. Hess, Muñoz Garm. & Kadereit) P.P. Ferrer, comb. & stat. nov. (see below), according to the new combination based on *Olea europaea* subsp. *guanchica* [6] [basionym].

**Figure 5:**
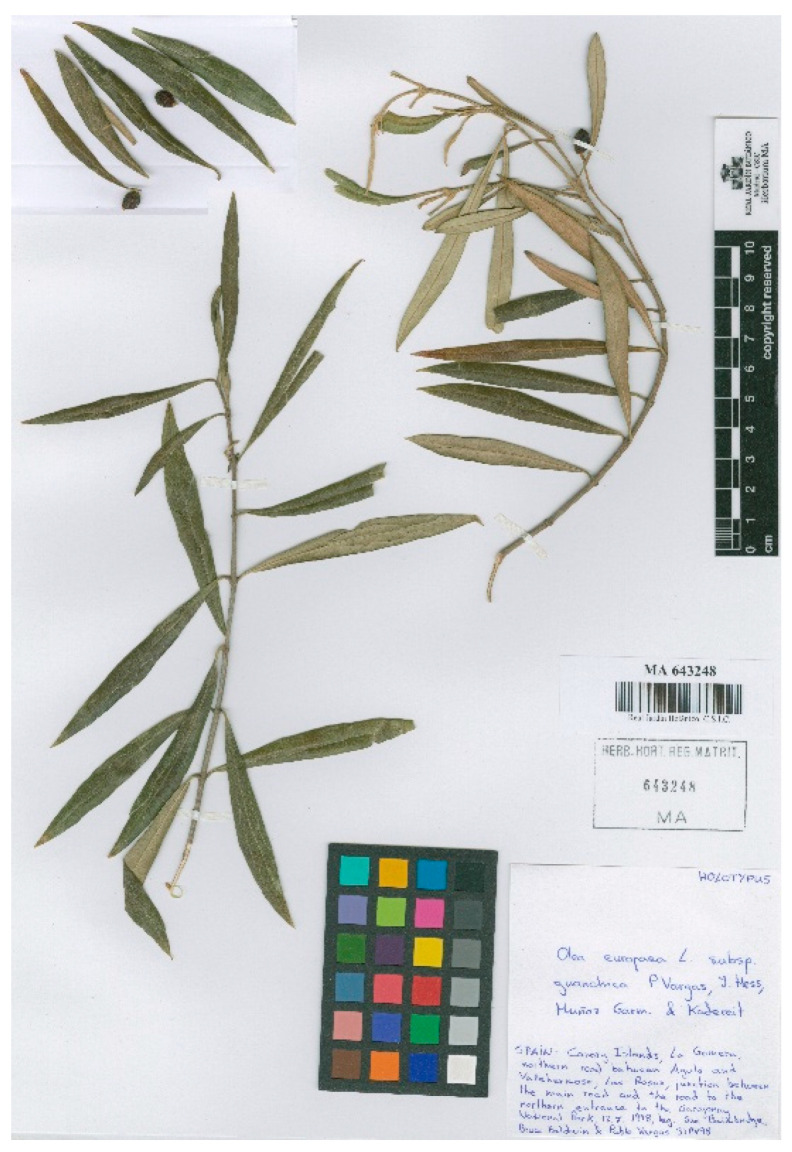
Holotype of *Olea europaea* subsp. *guanchica* P. Vargas, J. Hess, Muñoz Garm. & Kadereit, MA barcode MA 643248. Image courtesy of the herbarium MA, reproduced with permission.

***Olea europaea*** subsp. ***guanchica*** P. Vargas, J. Hess, Muñoz Garm. & Kadereit in Anales Jard. Bot. Madrid 58(2): 361. 2001 ≡ *O. cerasiformis* Rivas Mart. & del Arco in Itin. Geobot. 15: 705. 2002 ≡ *Olea guanchica* (P. Vargas, J. Hess, Muñoz Garm. & Kadereit) P.P. Ferrer, **comb. & stat. nov.**–Holotypus: Spain, Canary Islands, La Gomera, northern road between Agulo and Vallehermoso, Las Rosas, junction between the main road and the road to the northern entrance to the Garajonay National Park, 17 July 1998, *Su Baimbridge*, *Bruce Baldwin & Pablo Vargas 31PV98*, MA barcode MA 643248. Isotypus: MJG. For an image of the holotype, see Figure 5.

#### 2.2.2. *Olea cuspidata*

The protologue of *Olea cuspidata* [98] consists of an English description listed under the number “16”: “glabrous; leaves oblong-lanceolate, attenuated at both ends, cuspidate at the apex, rusty beneath; panicles terminal and axillary”, followed by de symbol “Ϧ”, indicating that the species is a shrubby, and the letter “G.” [likely “Greenhouse”], and the provenance “Native of Kamaon” [Kumaon region]. The protologue also includes a gathering, indicated as “(Wall. cat. not. 2817.)”. No specimen or gathering was designated as the type.

Green (page 95) [4] mentioned that a specimen at “K-W” is the “holotype” of the name: “*O. cuspidata* Wall. ex G. Don, Gen. Syst. 4: 49 (1837). Type: India, Kumoan, Wallich 2817 (holotype K-W)”. However, this specimen is not a holotype because several duplicates are known at K and G, all of which belong to the gathering cited in the protologue (i.e., “Wall. cat. not. 2817”), and therefore constitute syntypes. Although the selection of a duplicate at K as the lectotype would be appropriate, Green’s use of “holotype” cannot be corrected to a lectotypification because the phrase “here designated” (“*hic designatus*”) or an equivalent expression was not used, according to the International Code of Nomenclature (*ICN*) (Art. 7.11) [92].

**Figure 6:**
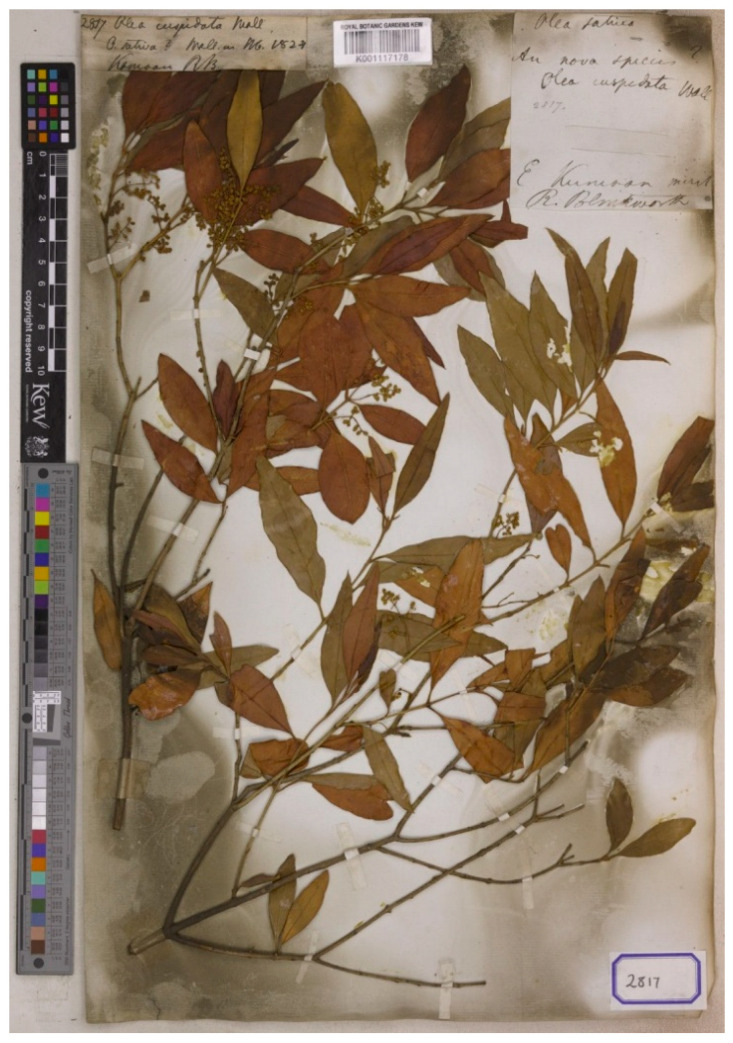
Lectotype of *Olea cuspidata* Wall. ex G. Don, K barcode K001117178. Image courtesy of the herbarium K, reproduced with permission.

We have found three relevant specimens preserved at G and K herbaria, with barcodes K000978905, K001117178, and G00383495. The sheet K000978905 bears a stem with leaves and immature flowers, and a handwritten label annotated as “2817 *Olea cuspidata* Wall/*O sativa*? Wall. in Hb. 1824/Kamaon RB” [Richard Blinkworth]. The sheet K001117178 bears several stems, with leaves and inflorescences with immature flowers, and two handwritten labels: (1) “2817 Olea cuspidata Wall/*O sativa*? Wall. in Hb. 1824/Kamaon RB” [Richard Blinkworth], and (2) “*Olea sativa*/In nova species?/Olea cuspidata Wall/2817/E. Kumaon/R. Blinkworth”. Finally, the sheet G00383495 bears a stem with leaves and inmature inflorescences, an envelope with three leaves, and two handwritten labels: (1) “Olea cuspidata Wall./O sativa? Wall. in Hb. 1824/Kamaon/Cat. No. 2817/M. Wallich, 1829”, and (2) “2817” (Figure 6).

These three specimens belong to the gathering mentioned in the protologue and can therefore be treated as syntypes. Richard Blinkworth collected this material in 1824 in “Kumaon”, a region within present-day Uttarakhand (India). Uttarakhand has traditionally been divided into two parts: the western part known as Garhwal and the eastern part called Kumaon. Richard Blinkworth was an English botanist contemporary with other leading botanists of his time, such as Joseph Dalton Hooker and Nathaniel Wallich. These three specimens are in good condition of preservation and represents the traditional concept and current use of the name. We prefer to designate the specimen K001117178 as lectotype of this name because is the more complete and element of the original material.

*Olea cuspidata* Wall. ex G. Don, Gen. Hist. 4: 49. 1837 ≡ ***Olea europaea*** subsp. ***cuspidata*** (Wall. ex G. Don) Cif., Olivicoltore Rivista Olearia Ital. 19(5): 96. 1942–**Lectotype (designated here):** [India] “Kamaon” [Kumaon, Uttarakhand], 1824, *Richard Blinkworth 2817*, K (barcode K001117178). Isolectotypes: K000978905 and G00383495. For an image of the lectotype, see Figure 6.

#### 2.2.3. *Olea laperrinei*

The protologue of *Olea laperrinei* includes the common name “Aleo des Touaregs” followed by the comment: “N’ayant pas eu d’échantillons complets de cet arbre nous ne pouvons en donner qu’une description provisoire”, and a description in French: “Arbre moyen à cime étalée, rameaux grêles, les pousses de l’année blanches recouvertes de poils en écusson, feuilles linéaires lancéolées avec un mucron très développé, argentées en dessous, de 30 à 45 mm. sur 4–6 mm” [Medium-sized tree with a spreading crown, slender branches, the current year’s shoots white and covered with shield-shaped hairs, linear-lanceolate leaves with a well-developed mucro, silvery underneath, measuring 30 to 45 mm in length and 4–6 mm in width], and also a diagnosis in French. The protologue also includes a provenance “Hab. Djebel Debnat à l’Est de Tamanrasset” and the comment “Duveyrier dit de l’Aleo: “Gran arbre, dit-on, en tout semblable ‘a l’Olivier à l’exception de son fruit qui n’est pas une olive, il se montre par petits groups dans quelques stations du Hoggar” (p. 212)”. No specimen or gathering was designated as the type [99].

Louis Duveyrier (1818–1892) was a prominent French explorer, geographer and ethnographer, known for his expeditions in North Africa, particularly in the Sahara. In his work “*Exploration du Sahara: Les Touâreg du nord*”, Duveyrier (1864: 212) [100] mentioned, in addition to what is stated in the protologue, the following: “*Aleo (temáhaq)./Je suis d’autant plus dispose à identifier l’aleo au Phylliræa que, d’après le rapport de Valentin Ferdinand, le Phylliræa existerait dans une île au Sud de celle d’Arguin sur la côte de l’Océan./Rien d’étonnant, d’ailleurs, de trouver cet arbre là où vivent le thuya et le laurier rose. L’altitude explique la presence de ces arbres dans ces stations méridionales*” [I am even more inclined to identify the *aleo* with *Phylliræa* because, according to the report by Valentin Ferdinand, *Phylliræa* occurs on an island south of Arguin Island along the [Atlantic] Ocean coast. Furthermore, it is not surprising to find this tree growing alongside thuja and oleander. The altitude accounts for the presence of these trees in these southern locations]. However, unfortunately, no illustration of this plant appears in this book.

Green (2002: 97) [4] mentioned a specimen at “AL” as the “holotype” of the name: “*O. laperrinei* Batt. & Trab., Bull. Soc. Bot. France 58: 626 & 672 (1912) [99], as “*laperrini*”. Type: Algeria, Haggar Mts, Laperrine (holotype AL?, n.v. [i.e., *non vidi*, not seen])”. However, this sheet is not a holotype because at least one duplicate is preserved at MPU (see below), which is part of the same gathering collected by Laperrine in the Massif du Hoggar, and corresponds to the material cited in the protologue as “dans quelques stations du Hoggar”. Although the selection of the specimen at AL as lectotype would be appropriate, Green’s use of “holotype” cannot be corrected to a lectotypification because the phrase “here designated” (“*hic designatus*”) or an equivalent expression was not used, according to the International Code of Nomenclature (*ICN*) (Art. 7.11) [92].

Among the original material, we have found two relevant specimens at MPU, with barcodes MPU006796 and MPU006797. The specimen MPU006796 consists of three parts, each with plant fragments mounted on separate sheet cuttings, all of which are included on a single sheet. The cutout mounted on the upper left side of the sheet contains seven small fragments of branches with leaves. It is annotated as “N°. 7”, and “Aliou/tete du l’oued Tifoudjidjine”, and in a different spelling “Olea/Alea/Laperrine”. The locality “l’Oued Tifoudjidjine” refers to a river or stream in the Sahara Desert, located in southern Algeria, near the Tassili n’Ajjer mountain range, which is the same region mentioned in the protologue “Djebel Debnat”. The second part of the sheet is mounted in the upper-right corner, containing a small branch fragment with leaves, and a cut-out at the top, with the words “Herbier Battandier” printed. The third sheet is mounted in the lower-left corner and contains two branch fragments with leaves. Overall, the sheet, where these three fragments are mounted, bears several labels, two of which are revision labels, along with a cutout of the protologue and another handwritten label that reads: “Université d’Alger/Herbier de l’Afrique du Nord/Olea Laperrinei Batt. et Trab.!/(Type!)/Sahara central: Ahaggar, tête de/l’Ouet Tifoudjidjine./leg. Dautheville./en Targui: ‘Aliou’/D. M. Maire”. Jean Dautheville (1850–1916), a French botanist and naturalist, is best known for his expeditions in the Sahara, where he collected and studied desert plants. Unfortunately, no collection date is mentioned anywhere on the sheet.

**Figure 7:**
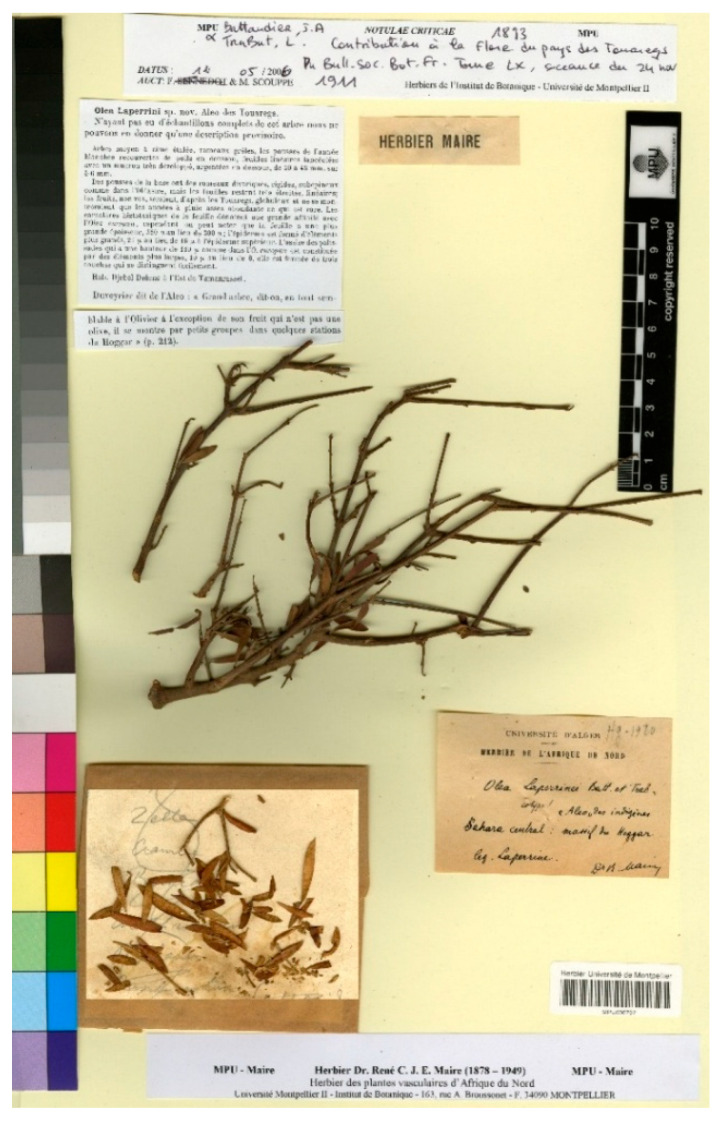
Lectotype of *Olea laperrinei* Batt. & Trab., MPU barcode MPU006797. Image courtesy of the herbarium MPU, reproduced with permission.

The second sheet, barcoded MPU006797, contains a specimen of this species, although it is in poor condition of preservation. The specimen consists of a branch with leaves, several smaller branch fragments, and an envelope containing remnants of leaves. This sheet also includes two revision labels, a cut from the protologue, and a handwritten label stating: “Université d’Alger/Herbier de l’Afrique du Nord/Olea Laperrinei Batt. et Tra./cotype! ‘Aleo’ des indigènes/Sahara central: massif du Hoggar/Leg. Laperrine./D.M. Maire” (Figure 7). This sheet, like the previous one, does not indicate a collection date. The locality, while roughly similar, does not exactly match the name mentioned in the protologue, “Djebel Debnat”. It appears that this material is attributed to a collection by Laperrine. Louis Auguste V. Laperrine (1860–1920) was a French military officer and explorer, primarily known for his role in the French colonial expansion in North Africa. Between 1908 and 1914, Laperrine conducted several expeditions and military missions in Algeria and Tunisia.

We have not been able to locate any other material that could be considered original for this name in any of the herbaria consulted (e.g., G, LY, P).

Unfortunately, the two specimens mentioned are in very poor condition. However, as noted in the protologue, the authors were only able to study incomplete material (“N’ayant pas eu d’échantillons complets de cet arbre nous ne pouvons en donner qu’une description provisoire” [Having not received complete specimens of this tree, we can only provide a provisional description]). It appears that the two specimens were collected by different authors: Dautheville for specimen MPU006796 and Laperrine for specimen MPU006797. Although both originate from the same general geographical area, neither corresponds exactly to the locality mentioned in the prologue “Djebel Debnat”. Furthermore, both specimens unfortunately lack a collection date.

Among these two specimens, we select MPU006797 as the lectotype of the name. Although the material is in poor condition, it is selected as the type because it is the only extant specimen directly linked to the original gathering cited in the protologue. No other original material has been located in the herbaria consulted, and this specimen provides the most reliable physical reference for the application of the name. Its selection ensures nomenclatural stability and allows future researchers to accurately identify and compare this taxon.

*Olea laperrinei* Batt. & Trab., Bull. Soc. Bot. France 58(2): 672. 1912 [‘*Laperrini*’] ≡ ***Olea europaea*** subsp. ***laperrinei*** (Batt. & Trab.) Cif., Olivicoltore Rivista Olearia Ital. 15(5): 96. 1942–**Lectotype (designated here):** Algeria, Sahara central, Massif du Hoggar, s.d., *L.A.V. Laperrine s.n.*, MPU (barcode MPU006797). For an image of the lectotype, see Figure 7.

#### 2.2.4. *Olea sylvestris*

The protologue of *Olea sylvestris* Miller (1768: *Olea* No. 3) [101] includes a Latin diagnosis “3. OLEA (*Sylvestris*) foliis lanceolatis obtusis rigidis, subtus incanis” and its English translation “Olive with spear-shaped, obtuse, rigid leaves, which are hoary on their underside”, followed by the synonym “Olea sylvestris, folio duro, subtus incano. C. B. P. 472.” (this reference being to Bauhin, 1623: 472) [102] and the common name “The wild Olive with a hard leaf, and horay on its under side”. A further description is also given in the main article on the genus *Olea*: “The third sort is the wild Olive, which grows naturally in woods, in the south of France, Spain, and Italy, so is never cultivated; the leaves of this sort are much shorter and stiffer than those of the other; the branches are frequently armed with thorns, and the fruits is small and of no value”.

Miller does not cite any specimens in the protologue, and the reference to Bauhin (1623) [102], included by Miller in the protologue, does not include any illustration for the polynomial “*Olea sylvestris*, folio duro, subtus incano”.

Among the elements used by Miller to describe his species, there is a relevant specimen in the general collection at BM which should be considered as original material for *Olea sylvestris*. The sheet, with barcode BM015176764, is currently filed at BM as *Olea europaea* L. It was collected by William Houstoun in Leiden Botanical Garden in 1728 (the base of the label is annotated “H. L. B. 1728” [i.e, *Hortus Lugdono Batavo* or Leiden Botanical Garden]), where Houstoun was a student of Hermann Boerhaave at that time.

Boerhaave (1727: 218) [103] includes this species (*O. sylvestris*) in his *Index alter plantarum quae in Horto Academico Lugduno-Batavo aluntur*, citing it as: “Olea T. 598. 320.” “2. Olea; sylvestris, folio duro, subtus incano. *C.B.P. 472. Oleaster*, *sive Olea sylvestris. I.B.* I. VI. 17. *Sylvestris Olea. Clus. H. 26. H.*”. A possible origin of cultivation of this plant in Leiden may be due to Clusius and his travels around the Mediterranean. The Leiden Botanical Garden, also known as *Hortus Botanicus Leiden*, is one of the oldest and most prestigious botanical gardens in the world. It is located in the city of Leiden, in the Netherlands, and was founded in 1590. The garden was further developed and expanded by the physician and botanist Charles de l’Écluse (in Latin, Carolus Clusius), who was hired by Leiden University in 1593 as a professor of botany. Under his direction (1593–1601), the garden grew significantly, initially focusing on medicinal plants and rare species.

Before becoming the director of the Leiden Botanical Garden, Clusius traveled to several Mediterranean countries. During these journeys, he studied and collected a wide variety of plants, many of which he later brought back to the Netherlands. These travels were crucial to his career, as they not only allowed him to establish valuable connections with other botanists and naturalists but also exposed him to new plant species that he would go on to cultivate and research in his garden. Clusius cites the olive tree “Olea Sativa” and the “oleaster or Silvestris Olea” in his *Rariorum plantarum historia* (Clusius, 1601: 25–26) [104] and includes an illustration of each species. Specifically for the oleaster he mentions the following: “*Nascitur quibusdam collibus juxta Tagum*, *& multis Lusitaniæ desertis confragosisque locis*, *inter ingentia saxa*, *præsertim non procul à terra postrema Lusitania (ut nunc circumscripta est) urbe*, *duobus supra Anam flumen miliariibus*, *ad radices Serrae Castulonensis saltus*, *quem nunc Sierra morena*, *hoc est Montem nigrum vocant*, *cujus pars superanda est eis qui Ulissipone Hispalim proficiscuntur*, *ubi magnae ejus sunt silvae*, *in quibus Ianuario mense fructum colligebant anno MDLXV. Maturus sit ejus fructus cum Olea*, *sed magna ex parte incolis negligitur*, *& in sturnorum aliarumque avium escam tantum cedit*” [trans: “It originates in certain hills near Tagum [the Tagus River?], and in many desert and rugged places of Lusitania, among huge rocks, especially not far from the last city of Lusitania (as it is now defined), two miles above the Anam River, at the foot of the pass of the Castulonensis Mountain Range, which is now called *Sierra Morena*, that is, the Black Mountain, a part of which must be crossed by those traveling from Lisbon to Seville, where its large forests are located, in which fruits were gathered in the month of January in the year 1565. Its fruit ripens alongside the olive, but it is largely neglected by the inhabitants, and is mostly left as food for starlings and other birds”].

However, there are other options. William Houstoun (1695–1733) was a Scottish physician, botanist, and naturalist. He studied medicine at the University of Edinburgh and later moved to the Netherlands, where he continued his studies at the University of Leiden from 1727. He was a student of Hermann Boerhaave. Houstoun traveled throughout Europe, collecting plant specimens and sending them to contemporary botanists across Europe. Houstoun himself likely collected this specimen in 1728, i.e., when he was a student there. It was then incorporated into his own herbarium. When Houstoun died in Jamaica in 1733, Miller inherited his herbarium and manuscripts, and when Miller died in 1771, Miller’s entire herbarium (including that of Houstoun) was bought by Sir Joseph Banks in 1774 and later was gradually incorporated into the Banksian Herbarium, which later became the BM.

Houstoun was a close friend of Miller, and when he (Houstoun) died in Jamaica in 1733, Miller inherited his entire herbarium collection (and also his manuscripts) [105,106]. The specimen barcoded BM015176764 must have been in Miller’s possession before 1768 and is certainly a good candidate for the lectotype.

**Figure 8:**
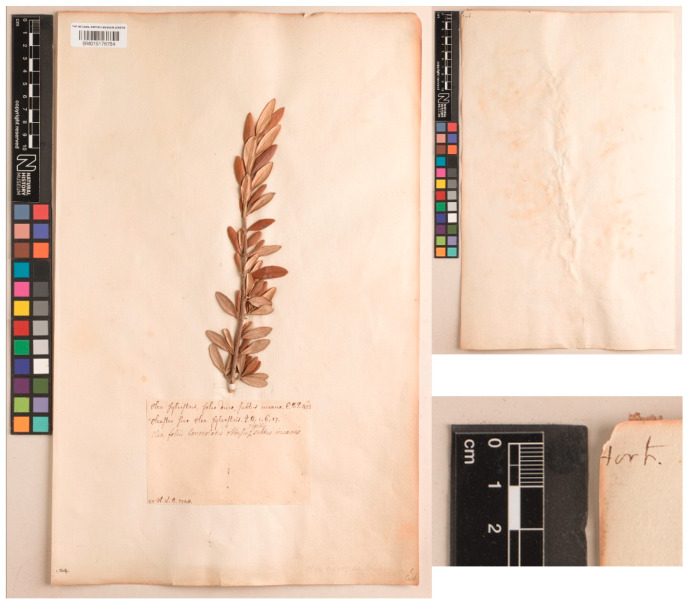
Lectotype of *Olea sylvestris* Mill. Specimen from a plant cultivated in Leiden Botanical Garden and collected in 1728 by Willam Houstoun, currently preserved at BM, with barcode BM015176764. Image courtesy of the Trustees of the Natural History Museum, London, reproduced with permission.

This relevant sheet, BM015176764, bears only a stem with leaves, and a label with three Latin polynomials (Figure 8). The first two lines are in Houstoun’s hand and the first of these is a Bauhin’s polynomial (“*Olea sylvestris*, *folio duro*, *subtus incano. C.B.P. 472*”) cited by Miller in synonymy of *Olea sylvestris*. In the second line, it is written: “*Oleaster sive Olea sylvestris. C.B. 1. 6. 17*”. The third line on the label is in Miller’s hand and has the Latin diagnosis of *Olea sylvestris*, the same as that in the protologue (“*Olea foliis lanceolatis obtusis rigidis*, *subtus incanis*”), so there is no doubt that Miller has seen and annotated this specimen, most likely before the 8th edition of the *Dictionary* was printed in 1768. The specimen does not have any prominent spines mentioned by Miller in the protologue, but it matches to description in many other respects, e.g., short, stiff (i.e., leathery) leaves, slightly pubescent on their undersides.

As this specimen was cultivated in Northern Europe, outside of its native range, it will be very difficult to ascertain where exactly it came from, but judging from Miller’s description in the *Dictionary* and from Clusius’ travels cited above, it probably originated somewhere in Southern Europe (France, Spain or Italy according to the Dictionary).

**Figure 9:**
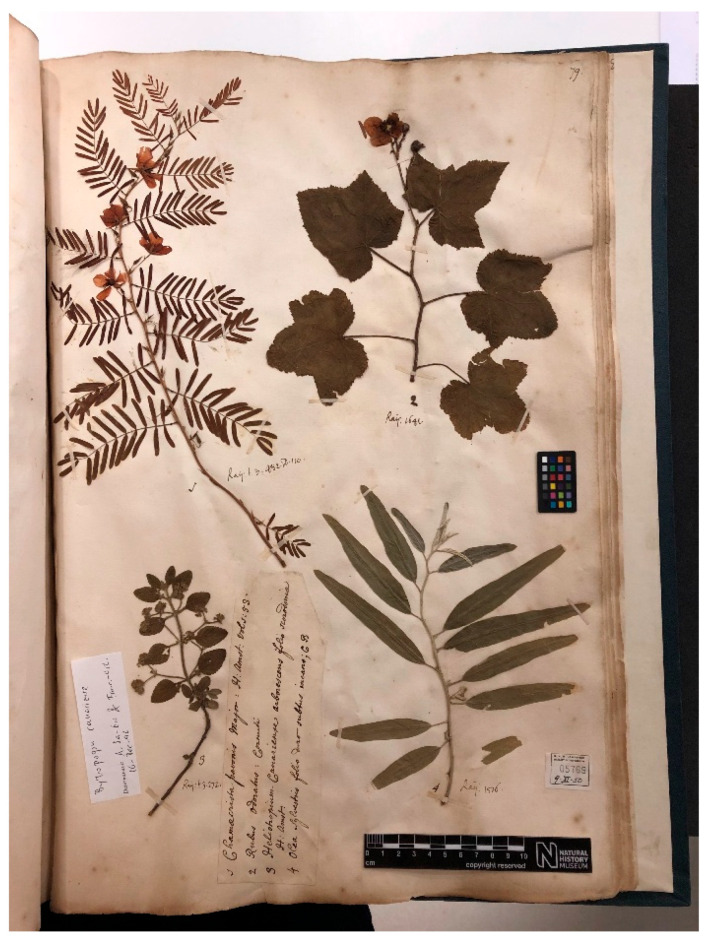
Herbarium sheet with a specimen of *Elaeagnus angustifolia* L. (H.S. 228 fol. 79.4) from a plant cultivated by Miller at Chelsea Physic Garden in the 1730s. Image courtesy of the Trustees of the Natural History Museum, London, reproduced with permission.

There is another relevant specimen in the Sloane Herbarium (kept separate from our main collection) which was cultivated by Miller at Chelsea Physic Garden in the 1730s and which Miller annotated with the same polynomial by Bauhin (i.e., “*4 Olea Sylvestris folio duro subtus incano; C.B.*”) as that he later cited in the synonymy of his *Olea sylvestris*. This specimen (H.S. 228 fol. 79.4), however, belongs to a completely different taxon, i.e., *Elaeagnus angustifolia* L. (Figure 9) [94]. This species, native to Central Asia, is sometimes confused with wild olives, as one of its common name, ‘Russian Olive’ implies. Miller described it as *Olea* No. 6 in the earlier editions of the *Dictionary* [107,108,109], but it was not included in the 8th edition in 1768 [101]. This specimen should therefore not be treated as original material for *Olea sylvestris*, but it is interesting to see that Miller was also tricked by its morphological appearance in ascribing it to the Olive genus at the beginning of his career.Regarding the material in the Sloane herbarium (H.S. 228 fol. 79.4; see Figure 9), according to Sloane (1707: 14) [110] “*Oleastri Species ut quídam putant*, *au alii Ziziphus alba. Gesn. hort. Germ. fol. 269*” is “one of the plants I gathered, or saw in the fields [in Madeira]” [Note: it seems Sloane had seen Oleaster in Madeira, but the H.S. 228 fol. 79.4 specimen was cultivated by Miller at Chelsea in the late 1720s or early 1730s]. In Madeira, *E. angustifolia* is not naturalized, and it is rarely cultivated [111], nor was it recorded for the archipelago by Lowe (1857–1868, 1872) [91,93,112]. Harcourt (1851: 137) [113] records this as *E. angustifolia*, a species found naturalized only in some areas of the island of Porto Santo [114,115]. According to Menezes de Sequeira et al. [111], it seems more likely that Sloane was referring to *O. maderensis*, a taxon that has narrower and more whitish leaves than the European species of *Olea*. However, the material preserved in the Sloane herbarium is undoubtedly a specimen of *E. angustifolia* as subsequently published by Santos-Guerra et al. (2020: 183) [94].

*Olea sylvestris* Mill., Gard. Dict., ed. 8: *Olea* No. 3. 1768 ≡ ***Olea europaea*** var. ***sylvestris*** (Mill.) Lehr in Diss. Bot.-Med. Olea europaea: 20. 1779 ≡ *Olea europaea* subsp. *sylvestris* (Mill.) Rouy ex Hegi, Ill. Fl. Mitt.-Eur. 5: 1936. 1927–**Lectotype (designated here):** [Specimen from a plant cultivated in Leiden Botanical Garden], 1728, *Willam Houstoun s.n.*, BM (barcode BM015176764). For an image of the lectotype, see Figure 9.

## 3. Materials and Methods

The designation of the types is based on the consultation of original material conserved in several herbaria and the literature cited in the respective protologues. Acronyms of the herbaria consulted are according to Thiers [116] (accesed on 22 January 2025). The identity of the designated types is verified with the current use of their respective names. The names in current use are set in bold italics typeface. The heterotypic synonyms are indicated with the symbol =, and the homotypic synonyms are indicated with the symbol ≡.

A relevant herbarium consulted for this study was the BM Herbarium, the herbarium of the Natural History Museum in London. This collection incorporates the original Sloane and Miller herbaria, which were later acquired by Joseph Banks, and are now preserved at BM [106,117,118].

All ICN (International Code of Nomenclature) Articles cited in the text refer to the Madrid Code [92].

## Data Availability

The original contributions presented in the study are included in the article, further inquiries can be directed to the corresponding authors.
